# Measuring the impacts of exposure to daycare quality on child development and nutrition measures through a large-scale randomised trial in Kenyan informal settlements: protocol

**DOI:** 10.1136/bmjopen-2025-112224

**Published:** 2026-03-09

**Authors:** Emily Beam, Anne Fitzpatrick, Maira Emy Reimão

**Affiliations:** 1 University of Vermont, Burlington, USA; 2 The Ohio State University, Columbus, USA; 3 Villanova University, Villanova, Pennsylvania, USA

**Keywords:** NUTRITION & DIETETICS, Child, Nutrition

## Abstract

**Introduction:**

High-quality early childhood education can fundamentally alter children’s long-term education, earnings and well-being. In low-resourced settings, children’s development is hampered by undernutrition, poverty and limited access to services. This study will generate high-quality evidence on child development from children who both attend and do not attend daycare across informal settlements in Kenya by (1) examining the relationship between child development, nutritional status and household characteristics using a large, community-based sample and (2) measuring the impact of exposure to improvements in childcare quality on child development and nutritional status.

**Methods and analysis:**

We combine a cross-sectional observational study of child development and anthropometrics among approximately 4700 children aged 0–5 across 11 counties in Kenya with a cluster randomised controlled trial that measures the impact of improved daycare quality on these outcomes. We use International Development and Early Learning Assessment to measure child development and record child height and weight to measure height-for-age and weight-for-height z-scores. Primary analyses will (1) estimate associations between child development outcomes and nutritional status using multivariable regression models, adjusting for prespecified covariates; and (2) examine differences in outcomes between children attending daycares in communities exposed to quality improvements and children attending daycares in control communities. Subgroup analyses will examine heterogeneity by child sex and daycare participation.

**Ethics and dissemination:**

The Strathmore University Institutional Scientific and Ethical Review Committee (SU-ISERC) has provided ethical review for this study, with initial approval SU-ISERC1602/23. This study has also received IRB approval from The Ohio State University (#2023B0300). Written informed consent will be obtained from caregivers before participation. We will disseminate findings through peer-reviewed publications, policy briefs and presentations to local stakeholders, and we will publish de-identified data and replication code on a public repository.

**Trial registration number:**

AEARCTR-0011747.

**STRENGTHS AND LIMITATIONS OF THIS STUDY**

- We use well-established methods and tools to measure nutritional status and early child development: we conduct direct anthropometric assessments using standardised field protocols, and we measure early child development outcomes using a validated, age-appropriate instrument (International Development and Early Learning Assessment) administered by trained enumerators.
- Data collection targets children aged 0–5 living in informal settlements across Kenya, a hard-to-reach population at high nutritional risk.
- Randomised assignment to differing levels of exposure to high-quality daycare provides a rigorous design to compare child outcomes while minimising confounding from provider and household characteristics.
- The sample is not randomly drawn from the informal settlements, and the sample of daycare users could not be recruited prior to randomisation due to high turnover in daycare enrolment.
- Due to the nature of the setting where measurements need to take place, collected data may be noisier, as some children who are being weighed may not be minimally dressed and the child development assessment may be conducted in a more crowded or distracting area than ideal.

## Introduction

### Background and rationale

 High-quality early childhood care and education is a determinant of children’s health, development and well-being, with strong evidence linking early environments to improved educational attainment, labour market outcomes and adult health.^1^,^3^ In many low-income and middle-income countries, including Kenya, such care remains scarce or unaffordable, especially for poor households.^4^,^7^ While Kenya substantially expanded public access to preprimary and primary school in recent years, families with young children rely heavily on the private market or informal arrangements for care.

In the urban informal settlements that house nearly half of Kenya’s urban population, paid daycare is largely informal and unregulated, with high child-to-caregiver ratios, limited caregiver training, few safe and stimulating materials, and insufficient opportunities for responsive and positive caregiver interactions.^8 9^ We find that 44% of daycares located in informal settlements across Kenya report having no toys and 37% have no books.^10^

Evidence from both high-income and low-income settings shows that structured and supportive interactions between caregivers and children are key pathways through which childcare quality influences developmental trajectories.^11^ Providers who are responsive, nurturing and cognitively stimulating promote secure attachment, language development and early learning skills,^12^ while low-quality childcare can constrain children’s socioemotional development and school readiness.^13^ Early childhood nutrition is also fundamental to child development, learning and future academic achievement.^14^ Malnutrition—whether due to insufficient calories, poor dietary diversity or micronutrient deficiencies—can impair brain development, reduce attentiveness and weaken immune function.^1 15^ In informal settlements in Kenya, approximately one-third of residents live below the poverty line, in conditions associated with malnutrition and micronutrient deficiencies.^16^ Roughly half of young children in Nairobi informal settlements are stunted,^17 18^ and we find that 40% of daycares report that at least one child spends the day hungry at least once per week.^10^ Informal daycare attendance may be associated with worse nutrition status,^19^ but this relationship may not be causal if malnutrition is correlated with enrolment decisions. Given this context, it is *a priori* unclear whether attending daycare is positively (or negatively) associated with health outcomes for children in informal settlements.

Although this population is highly vulnerable, data on child development and nutritional status in informal settlements are extremely limited. Families living in these settlements are mobile and hard to reach, making it particularly difficult to collect high-quality data.

We address this gap by (1) documenting child development and nutritional status for approximately 4700 young children in 51 informal settlements across Kenya and (2) assessing the causal impact of improvements in childcare quality on child development and anthropometric measurements for children enrolled in paid daycares within these settlements. A reliable, high-quality measurement of early childhood cognitive and physical development outcomes among a low-income population in Kenya is fundamental for policy design, as is an understanding of the effect of efforts to improve daycare services available to them on these outcomes.

In this study, we build on an existing cluster randomised controlled trial (C-RCT) that has been underway in informal settlements in Kenya since 2024 and involves nearly 1000 daycare firms.^10^ We will collect and analyse data on child development for children aged 3–5 (roughly 2700 children) and child anthropometric measurements for children aged 0–5 (roughly 4700 children) in the same communities where these daycares operate. The intervention within the C-RCT focuses on improving the quality of paid daycare, including improvements in caregiving practices, infrastructure and the provision of nutritious food. The study sample includes (a) young children from households enrolled in the study before the intervention and (b) young children currently enrolled in centres located in treatment and control communities.

### Study objectives

1. To measure early child development in children 3–5 living in informal settlements in Kenya through the International Development and Early Learning Assessment (IDELA) and compare our results to existing measurements of child development outcomes found elsewhere.
2. To assess the prevalence and severity of nutritional status deficiencies for children aged 0–5 living in informal settlements across Kenya through anthropometric measurements.
3. To determine the correlates of both early child development and nutritional status, including whether paid caregiving arrangements are associated with children’s outcomes.
4. To measure the causal impact of improvements in childcare quality on early child development and anthropometric measurements of nutritional status.

## Methods: participants, interventions and outcomes

### Study background

The protocol described here builds on an ongoing C-RCT by (1) including a new sample of children enrolled in our study daycares and (2) collecting new data on anthropometric measurements and direct assessments of child development. The existing C-RCT measures the impact of a social franchising model designed to improve childcare quality on firms and families.^10^ We partnered with a Kenya-based social franchising organisation that supports and facilitates the improvement of existing daycare centres through training and continuous mentorship on various areas relevant to running a daycare, including high-quality caregiving practices, health and safety, and business management. The intervention also includes a facility improvement grant and is broad, with components that could improve child health and nutritional status both directly and indirectly. First, the organisation requires and supports improved sanitation practices (eg, installing wash stations, separating toileting from eating locations, etc). Second, it works to improve the physical infrastructure, such as providing rugs for floors. Third, it provides subsidised, fortified porridge for children. These intervention components may directly improve nutritional status and increase cognitive development of children through improved engagement and nutrition.

For the C-RCT, we randomly assigned 25 communities to receive the treatment and 26 to be in the control group, stratifying by county. The research team conducted this randomisation independently after the firm listing, firm baseline and household listing were complete, at which point it shared the assignment with the implementing partner. In treatment communities, the social franchising organisation offered its programme to all existing daycare firms, and it trained and enrolled those interested in its services. The organisation did not enter control communities, allowing us to compare impacts in areas with and without exposure to the franchising model. In this study,^10^ we measure the impact of this social franchising model on childcare quality, provider revenues and parental work outcomes in urban informal settlements across Kenya.

**Figure 1:**
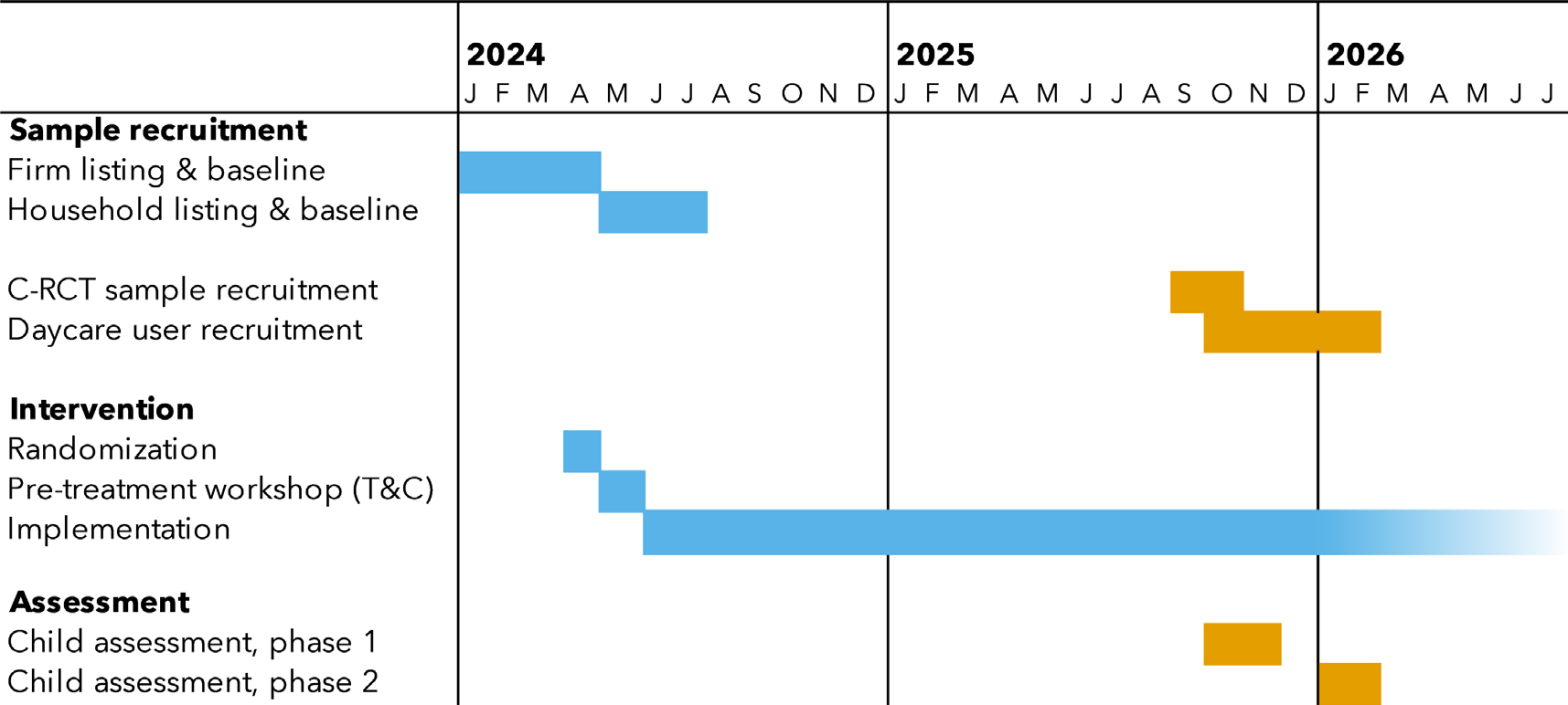
Study timeline. The figure shows timing for firm and household listing and baseline, randomisation and intervention, and two phases of child assessment. C-RCT, cluster randomised controlled trial. T&C, treatment and control.

**Figure 2:**
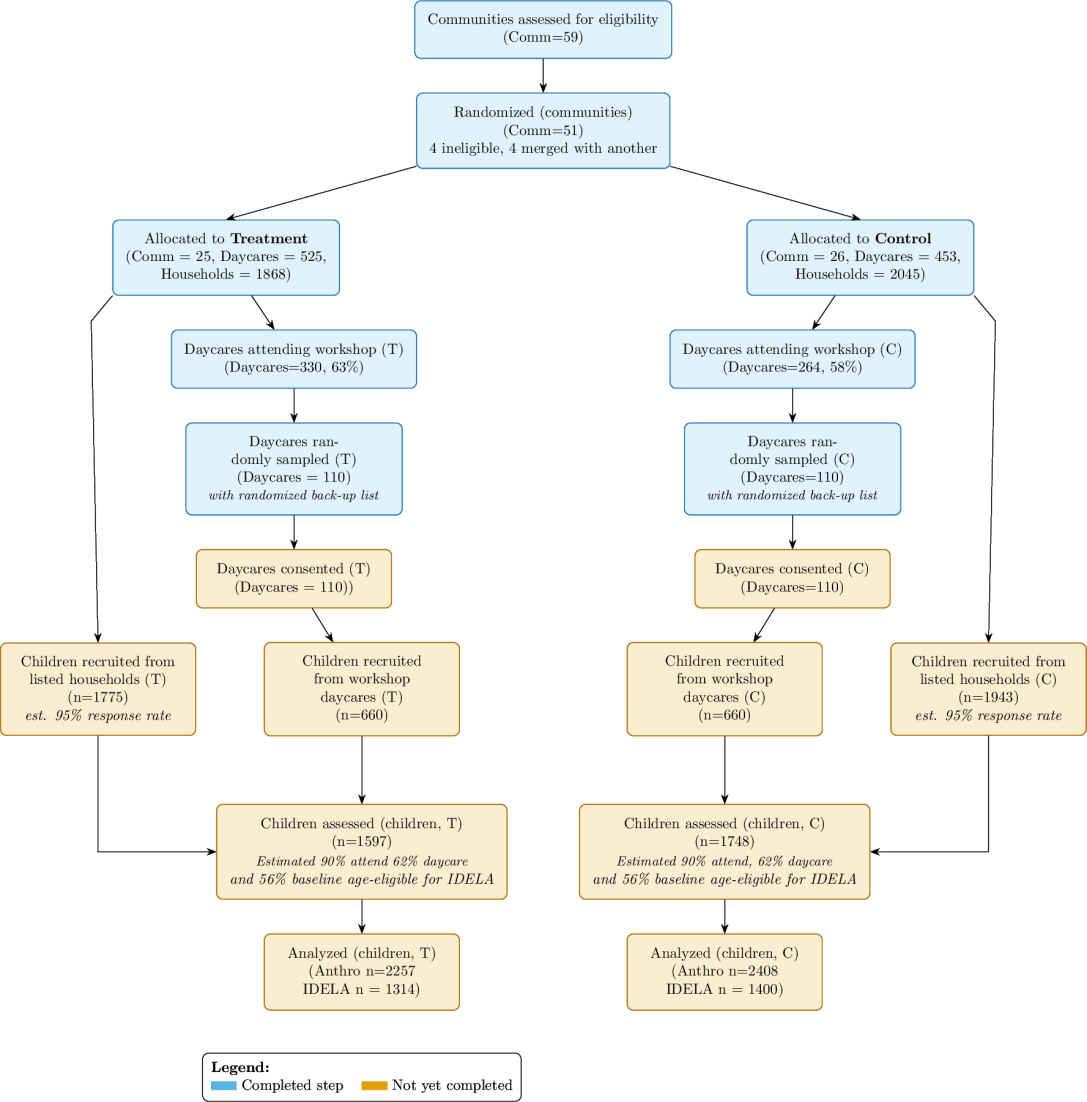
CONSORT diagram. The figure shows the selection and randomisation of communities, along with planned child recruitment from both the baseline sample and workshop daycares. CONSORT, Consolidated Standards of Reporting Trials Statement; IDELA, International Development and Early Learning Assessment.

In this protocol, we outline the procedures and expected contribution of the proposed study, which builds on this existing C-RCT. The overall study timeline is shown in figure 1, and the Consolidated Standards of Reporting Trials Statement (CONSORT) diagram is shown in figure 2. For this addition, we recruit children currently enrolled in daycare centres in both treatment and control communities, but who may not have been in our original study sample (as the sample frame for children in the latter comes from the community and will include both children who participate in daycare and those who do not). The current study focuses on children—rather than daycare firms or households—to measure child development and nutritional status among children who do and do not attend daycare in the study communities, and it compares outcomes between treatment and control communities.

### Study setting

We conduct our study in 51 low-income urban informal settlements located across 11 counties in Kenya. In early 2024, we first carried out an exercise to define the boundaries of the study communities, and therefore potential daycares and children living within them. With guidance from local leaders, study team members walked around the communities and outlined polygons using GPS that would correspond to cluster boundaries for each community. The boundary area was designed to be large enough to include a minimum number of eligible daycares; mapped boundaries were then adjusted accordingly to ensure that each community had at least 10 operating daycares.

### Firm recruitment

Within the 51 communities, we conducted a census of all paid daycares, and then a baseline survey with a subset in early 2024, reaching 978 providers. Our implementation partner then invited surveyed firms in treatment and control communities to attend a free half-day child development workshop. We denote the roughly 60% of baseline firms that attended this workshop as the ‘workshop sample’ (see Fitzpatrick *et al*^10^ for more details on their characteristics). In treatment communities, 82.5% of the workshop sample then opted to continue the partner’s programme, undergoing quality improvements and further training. Workshop-attending firms are comparable between treatment and control communities across the range of characteristics we consider, thus allowing us to use the ‘workshop sample’ to compare children in daycares that joined the social franchising organisation in treatment communities with children in daycares that would have likely joined the programme if the implementation partner had entered their community.

### Child recruitment

Children born in 2021 or later will be recruited from two sources: (1) households listed in the C-RCT baseline and (2) children currently enrolled in selected daycares. We recruit C-RCT baseline households following the midline survey, after enumerator trust has been built. For the daycare user sample, we ask providers to send study information and consent forms home with parents, and we send enumerators to directly recruit during drop-off times. See detailed recruitment procedures in online supplementalappendix A.1.

### Data collection

Enumerators will conduct assessments with these two samples of children, measuring height and weight for all children aged 0–5 to measure stunting and wasting and applying the IDELA for children aged 3 and older.^20^ These assessments will be carried out at local daycares (with agreement from daycare owners) and community centres. Measurements of height and weight will follow the 2019 WHO/UNICEF guidelines,^21^ and IDELA will be implemented following the tool guidelines. Details are provided in online supplemental appendix A.1.4 and A.1.5, respectively.

Data collection will be done in two stages, one in October 2025 and one in January 2026. (The two-stage split is because schools in Kenya are generally closed from November to January). Each stage of data collection will be done over 2 days in each community, with two enumerators per day.

All instruments will be programmed into SurveyCTO and administered using tablets. Enumerators will carry manipulatives and other materials as necessary.

#### Child age data

Our study sample is limited to children born in 2021 or later, and we will use child date of birth to calculate height-for-age z-scores (HAZ) as well as to apply the IDELA, which relies on child age in months. For our first group of children (those in the C-RCT baseline), we have the child’s month and year of birth, as that was recorded in the C-RCT baseline. This information will be verified during our midline survey, when parents are invited to bring their children for the assessment.

For the group of children recruited through their daycare participation, parents will be asked to provide the date of birth of their children in the forms they return to the daycare along with their consent.

#### Sample size and power calculations

As figure 2 shows, we plan to collect anthropometric measurements from 3345 baseline sample children (1748 control and 1597 treatment) and 660 daycare user sample children (330 control and 330 treatment). This yields 80% statistical power to detect a treatment effect of 0.14 SD on anthropometric measurements for each subsample. For child development, we have 80% power to detect a treatment effect of 0.25 SD, reflecting that roughly 60% of children will be in the relevant age range and that the estimated intra-cluster correlation coefficient is likely to be higher. See online supplemental appendix A.2 for more details. These minimum detectable effect sizes are well within the range of measured impacts found from similar interventions (see Dulal *et al,*^22^ development impacts ranging from 0.30 to 0.52 SD; and see Mamun *et al*,^23^ nutrition impacts averaging 0.20 SD).

### Oversight and monitoring

#### Data monitoring and quality

Data collection and implementation will be monitored by the research team. Innovations for Poverty Action-Kenya (IPA) will implement all data collection. IPA conducts real-time monitoring of data collection progress to track consent, response rates, refusal reasons and survey duration. During the data collection, IPA also carries out high-frequency data checks to identify potential data problems quickly, such as unexpected missing values. Research assistants and field supervisors also hold weekly debriefings to address any data quality or collection issues in real time. Further details on enumeration team training can be found in online supplemental appendix A.1.3.

The child development assessments will follow data quality guidelines. Supervisors will sit in and observe a subset of child assessments and ensure that all ethical and protocol guidelines are being strictly followed. During the IDELA assessment, there are two questions where children are asked to make drawings or write on paper. Enumerators will photograph their work for a randomly selected 10% sample, which will be reviewed by another staff member to ensure accurate scoring. For anthropometric measurements, data collection will follow the 2019 WHO/UNICEF guidelines,^21^ with details in online supplemental appendix A.1.4. IPA also uses the data quality monitoring checks built into SurveyCTO.

Because this intervention and data collection pose minimal risk, we do not plan external monitoring or formal external auditing.

#### Harms

Because this study comprises data collection with young children alongside a minimal-risk intervention delivered to childcare providers, we do not anticipate any harms, though study participation could disrupt providers’ daily care routines, and children may not want to participate in the assessment. Field staff will prebook assessment sessions to minimise inconvenience to providers, and they will be trained to identify signs that a child is upset or does not assent to participation. IPA will document any adverse events and share them with the PIs. Any serious incidents will be reported promptly to the institutional review boards overseeing this study.

### Participant and public involvement

The participants and/or the public were not involved in the design, conduct, reporting or dissemination plans of this study.

## Statistical analysis

Our primary outcomes are anthropometric indicators (height and weight) of child nutrition and child development indices across cognitive, socioemotional, motor and language domains.

### Primary outcomes

#### Nutritional status

- HAZ.
- Weight-for-age z-scores.
- Stunting, wasting and underweight.

These will be calculated using WHO growth standards.^24^

#### Child development

- Domain-specific scores of IDELA (motor development; emergent literacy; emergent numeracy; socioemotional development) standardised to age-adjusted z-scores using the reference distribution.
- Composite index of child development created using item response theory.

### Data analysis

First, we will analyse and produce descriptive statistics of the key child development outcomes listed above. Second, we will compare these measurements for children in the treatment communities to those in control communities according to the larger study’s randomised design using multivariable regression. We will include controls for child age, sex and strata (county) fixed effects.

We will also examine these outcomes separately for the C-RCT baseline sample and daycare user sample. Because C-RCT baseline households were identified prior to randomisation and the intervention, we anticipate that differences in outcomes will reflect the causal intention-to-treat impact of improved childcare in the community. For the daycare user sample, we will capture the treatment-on-treated effect, but this could be biased if the quality improvement affected the selection of children into daycares.

We will restrict our child development analysis to children for whom we obtain IDELA data. Furthermore, we restrict our nutritional status analysis to children for whom we obtain valid anthropometric measurements. In the event of missing independent variables, we will code them as zero and include a missing variable flag.

Cross-sectional data gathered concurrently through caregiver and daycare firm surveys will produce a large dataset for measuring the relationship between early child development and nutritional status, as well as whether paid caregiving arrangements are associated with children’s health and development outcomes.

The final report of this study will follow the general CONSORT.

#### Subsample analysis and heterogeneity

We will examine effects among our two different sample sources: the children recruited through our C-RCT household sample and those recruited from among children currently enrolled in the selected 220 daycares. We will also conduct subgroup analyses along the following dimensions:

- Daycare enrolment status: whether using daycare or not; type of daycare used (home and centre vs school-based daycare) and quality of daycare used.
- Child sex.

### Software

Data will be collected electronically using SurveyCTO Collect V.2.81 on Samsung Galaxy Tab A7 Lite tablets with Android V.12. All statistical analyses will be carried out in Stata V.18 or newer.

## Ethics and dissemination

### Research ethics approval

The Strathmore University Institutional Scientific and Ethical Review Committee (SU-ISERC) has provided ethical review for this study, with initial approval SU-ISERC1602/23. This study has also received IRB approval from The Ohio State University (#2023B0300). The University of Vermont Committee on Human Subjects determined the UVM-affiliated activities to be exempt human subjects research under 45 CFR 46.104(d)(4) (STUDY00002790, 1 November 2023); UVM is not the reviewing IRB for the overall study. Significant amendments will be approved by both Strathmore and OSU IRBs, updated in the trial registry and communicated to partners and funders. Because this study has minimal risk, we do not have a data monitoring committee and do not have stopping guidelines in place.

Trial conduct will be monitored internally by the principal investigators and project management team. Field supervisors will conduct routine spot checks to ensure adherence to study protocols, participant confidentiality and data quality standards. Data will be reviewed weekly for completeness and consistency, and any deviations from protocol will be discussed and documented during biweekly implementation meetings. Given the minimal risk nature of the intervention, no independent monitoring body is deemed necessary.

### Consent and assent

We will only collect measures from children for whom we have received informed consent from their caregiver. For children whose households are in the C-RCT survey sample, we will request consent from caregivers to measure and assess their children at the conclusion of the caregiver interview. The child assessment will take place at a separate point in time, and caregivers will be invited to bring the child to that appointment. For the daycare user sample, we will work with daycare owners to obtain informed consent from the caregivers of the children enrolled in their daycare.

Child assent: Once parental consent has been obtained, enumerators will seek child assent before starting the assessment and throughout the process. Assent will be interpreted broadly, with refusal including either verbal or physical hesitancy. If a child becomes very upset, scared or angry, the enumerator will record that the child did not assent and move on to the next child.

### Confidentiality

Consistent with our IRB-approved protocol, we will use appropriate data safeguards and precautions at all stages of the research to ensure that identifiable data are kept securely and not shared with individuals outside of the study team.

### Child safeguarding

IPA has developed a rigorous child safeguarding policy that governs this data collection exercise. All team members will be trained on the care and conduct of research with children, and behaviour will be monitored for compliance.

### Dissemination

We will share study findings through presentations to stakeholders and policymakers, policy briefs and blog posts. The research team will prepare and disseminate academic papers and present at national and international conferences. Following publication, we will publish replication code and deidentified datasets in a public repository.

## Supplementary material

10.1136/bmjopen-2025-112224 online supplemental file 1
