# Supplementary material for: Measuring the impacts of exposure to daycare quality on child development and nutrition measures through a large-scale randomised trial in Kenyan informal settlements: protocol
Source: BMJ Open. 2026 Mar 9;16(3):e112224. doi: 10.1136/bmjopen-2025-112224 (PMC12983927; doi:10.1136/bmjopen-2025-112224)
Supplement: online supplemental file 1 [file bmjopen-16-3-s001.docx]

# Supplementary Appendix

## A.1 Child recruitment

Children in the current study, for whom we will measure child development and nutritional status, come from two sources: a listing sample, which is a sample of households that were identified in 2024 as living within the study community boundaries, and a daycare user sample, which includes children currently enrolled in “workshop sample” daycares.

### A.1.1 Listing sample

We generated a “listing sample” of households in 2024 by canvassing the study communities to identify and recruit households with children born during or after 2021. We listed 5,741 households within the study communities, of which 5,633 were eligible for participation in the baseline survey. Households were recruited through various methods, including door-to-door visits, approaching individuals in community markets, and at local daycare facilities.

For our C-RCT, we randomly selected 3,419 of these households for baseline in April–July 2024, completing the survey with 3,029 of them. This baseline survey included information on parental labor force participation, daycare use, and caregiving practices. However, that data collection round did not include a caregiver-reported child development module, direct child development assessments with children, or the collection of child anthropometric measurements.

In September–October 2025, we will survey with the households in the baseline and the remaining 2,214 listed households that were not surveyed at baseline. For the current study, at the end of the household survey, we identify one child born in 2021 or later in each household as the “target child.” This child will typically be the youngest in the household at the time of the baseline survey (2024). However, if that child is no longer in the household, we will replace them with another child in the household born in 2021 or later. (If there are no other children in that age range, we consider a child born in 2019 or more recently; if the household does not have any child born in 2019 or more recently, we remove them from the sample.) The caregiver of this child (generally the respondent to the household survey) will then be invited to bring the child at a pre-determined time and location within the community for anthropometric measurements and, if the child is aged 3 or older, a direct enumerator-administered child development assessment, as described in the Data collection section of this protocol.

In the September–October 2025 household survey round, we will use the GSED short-form to collect caregiver-reported indicators of child development for the same children who are invited for the in-person assessment. The GSED tool was originally available in English. A member of the team first translated it into Swahili, and the survey was back-translated and rigorously tested by multiple study team members. The GSED results do not fall within the main objectives of the current study, though they may be used to assist in our analysis and interpretation of the direct child assessment.

### A.1.2 Daycare user sample recruitment

Additionally, we will select four daycare firms from each of the 51 communities. There are an additional 8 daycares selected in four treatment communities because these communities were merged after mapping but before randomization, yielding a total of 220 daycares. We target daycares that participated in the 2024 pre-treatment workshop (i.e., they are in the “workshop sample”). These daycares are comparable between treatment and control communities on baseline characteristics and reflect the set of likely takers. That is, if they are in a treatment community, they are significantly more likely to have participated in the quality-improvement intervention than non-attenders. And if they are in a control community, they would have been highly likely to participate had the program been offered in their community. In the event a community does not have enough firms that either agree to participate in the daycare user sample, or a sufficient number of children within the relevant age range enrolled, additional firms within the sample will be recruited as replacements.

All children born in 2021 and later enrolled in the selected daycares will be invited to participate in the height and weight measurement and, if aged 3 and older, the enumerator-administered child development assessment. We will work with the daycares to secure caregiver consent, sending forms home with information on the assessment day and collecting parent signatures at drop off or pick up as relevant. During assessment days, all children who are present and whose parents provided consent will be invited to participate.

Note that daycares hosting these activities consist of both daycares that participated in the C-RCT treatment and those that did not. Enumerators are trained to not refer to the intervention or experiment in any discussion with respondents or caregivers.

### A.1.3 Piloting and team training

The procedures were rigorously piloted in three different counties of Kenya in communities outside of the study area in August–September 2025. Training for the full data collection effort will include 7 days of training for a team of 22 enumerators in September 2025, and an additional refresher training for at least 4 days in January 2026. The training agenda will include both classroom exercises and field practice, as well as safeguards for protecting child respondents.

### A.1.4 Anthropometric measurement

1. *Guardian and caregiver consent.* Enumerators should only collect height and weight measurements for children whose guardian previously consented. For children whose caregivers brought them to the measurement location, this will be obtained in written form from the caregiver at the time of the meeting. Information on the child’s date of birth will be verified when the caregiver is invited to bring the child for measurement.

For children who attend the daycares in which the assessments are conducted, consent will be obtained in advance, with participant daycares collecting the forms from caregivers and passing them onto the enumeration team. The forms given to caregivers and returned to the daycare will also require information on the birth date of the child. This information will not be verified through official documentation, as the assessments will be done with caregiver consent but not necessarily their presence at the daycare. If the caregiver provides consent but incomplete information on birth date, the enumerators will mark the incompleteness and proceed with the assessments.

1. *Preparation of children for height or length and weight measurements.* Once all permissions are obtained, enumerators will begin to prepare children for the measurement. As per WHO guidelines, children under 24 months are measured by length; children 24 months and over are measured by height. For the collection of height and length, children will remove their shoes. If the child’s hair would potentially interfere with the height measure, and the hair is arranged in a temporary fashion, then the hair will be taken down to allow for accurate measurement. Otherwise, if the child’s hair ornamentation would interfere with measurement, enumerators will be trained to collect the height or length measure from the top of the head.

- Children will next remove their sweaters, jackets, and shoes in an attempt to have the child wear “minimal clothing.” If the parent or legal guardian or daycare teacher is present, the enumerator may ask them for assistance. If a parent or legal guardian is not present, it may not be possible to have the child wear minimal clothing. Instead, enumerators will enter on the form which clothes the child is wearing, and, following the 2019 WHO/UNICEF guidelines, note that the child was “not undressed to the minimum” [22].

1. *Measurement of child height or length and weight.* Enumerators will measure both height or length and weight for one child before moving to the next child. Note that it takes 2 enumerators per child. For measuring child height/length, we use wooden height/length measuring boards approved by UNICEF, with a graduation of 0.1 cm and accuracy of 0.2 cm. Child height or length will be recorded in centimeters to the nearest decimal.

For child weight, we use a portable battery-powered digital scale^[[1]](#footnote-1)^ with a range of 0 to 150 kilograms and a graduation of 100g, and we follow the 2019 WHO/UNICEF recommendations for calibration. Child weight will be recorded in kilograms.

- For children who are too young to stand on the scale independently, we will first ask the caregiver to hold the child and collect the combined weight. Then, the caregiver will step on the scale without holding the child.

Biologically implausible values will be flagged and excluded prior to analysis following WHO-recommended cutoffs: height-for-age z-score < −6 or > +6; weight-for-age z-score < −6 or > +5; and weight-for-height z-score < −5 or > +5 [22]. Exclusion rates will be reported

Our primary results will use the initial height/length or weight measurement, following WHO guidelines. For robustness, we will also report estimates that average the initial estimate with a second estimate. In the case of substantial discrepancies (greater than 0.1 kg in weight or greater than 1cm in height/length), we will collect a third estimate and use the average of the two closest measurements.

### A.1.5 IDELA measurement

Enumerators will assess child development using the IDELA for children aged 36 months and up whose caregivers have provided consent. Training for the tool follows the materials provided by IDELA.

One enumerator can conduct IDELA with one child at a time. To the extent possible, they will work with the child in a quiet place away from distractions, including other children, within the assessment location.

## A.2 Power calculations

We calculate statistical power separately for the baseline and daycare user samples.

For the daycare user sample, we estimate a sample size of 1,320 children aged 0–5. Based on firm baseline data, we anticipate that 62% will be aged 3–5. For the household baseline sample, we have a frame of 2045 treatment households and 1868 control households with age-eligible children. We estimate that 56% will have target children aged 3–5, reflecting midline survey progress to date. We anticipate that 85.5% of the sampling frame is likely to bring their children for evaluation, reflecting the 95% response rate to date and field staff estimates of a 90% assessment attendance rate. (99% of parents surveyed so far have consented to the later assessment.)

Because we randomized partner entry at the community level, we must account for the potential correlation of child-level outcomes within communities. For anthropometrics, we conservatively use a community-level ICC of 0.03, based on [8]’s finding that most anthropometric measures had community-ICCs below 0.03 before covariate adjustment. Community-level ICCs on child development are less commonly available ([8] provides community-level ICCs averaging 0.2 for enrollment and attendance only). We use an ICC of 0.10, reflecting the additional likely variation in development outcomes vs. binary enrollment and attendance outcomes.

1. Other large-scale field research carried out in Kenya has used low-cost digital scales rather than high-precision scales, as the latter are better suited for central weighting, such as at a health-center, rather than weighing at each household or daycare, as in the present study. Additionally, low-cost digital scales are more manageable for field workers to carry around, an essential feature in this setting. To ensure reliability of the scales, we calibrate them on a weekly basis and replace any faulty ones. [↑](#footnote-ref-1)
