# Supplementary material for: Measuring the impacts of exposure to daycare quality on child development and nutrition measures through a large-scale randomised trial in Kenyan informal settlements: protocol
Source: BMJ Open. 2026 Mar 9;16(3):e112224. doi: 10.1136/bmjopen-2025-112224 (PMC12983927; doi:10.1136/bmjopen-2025-112224)
Supplement: Supplementary file 2 [file bmjopen-2025-112224.reviewer_comments.pdf]

PEER REVIEW HISTORY

BMJ Open publishes all reviews undertaken for accepted manuscripts. Reviewers are asked to complete a checklist review form and are provided with free text boxes to elaborate on their assessment. These free text comments are reproduced below.

ARTICLE DETAILS

Title (Provisional)

Protocol for measuring the impacts of exposure to daycare quality on child development and nutrition measures through a large-scale randomized trial in Kenyan informal settlements

Authors

Beam, Emily; Fitzpatrick, Anne; Reimão, Maira Emy

VERSION 1 - REVIEW

**Reviewer**	**1**
**Name**	**Kac, Gilberto**
**Affiliation**	**Rio de Janeiro Federal University**
**Date**	**12-Dec-2025**
**COI**	**None**

This is an interesting protocol that describes a study on child development from children who both attend and do not attend daycare across informal settlements in Kenya by (1) examining the relationship between child development, nutritional status, and household characteristics using a large, community-based sample and (2) measuring the impact of childcare quality improvements on child development and nutritional status.

I would like to highlight a few aspects to be considered by the authors, but the main one relates to how anthropometry is being handled in the study.

1. Page 6, line 36. For sake of clarity, I commend authors to find a better way to refer to anthropometric status. Currently at some instance is redundant or unclear the meaning. There is no reference to indicators such as stunting/wasting or underweight, that would make it much more clear to the readers.

2. There are important gaps/uncertainties on how anthropometric measures were collected. It is very important that authors adhere to what WHO/UNICEF recommends. Please consult their 2019 guideline. For example, authors should provide details about valid anthropometric. I am very concerned with the statement about using 4cm to flag differences between two measures. Also, can authors inform what is the range for defining outliers?

3. Additionally, please inform more clearly about date of birth, a key component to calculate anthropometric variables. I strongly recommend authors include a data quality section to clarify the questions I have made

4. Please state what type of equipment's are being used along with their brands and accuracy.

5. A few other aspects deserver attention.

a. Can authors write in full what C-RCT stands for in the abstract

b. it would be preferable to state what are the validates instruments here. Perhaps remove less important details from the abstract. I see intro is rather lengthy

c. I recommend the exposure is always listed before to ensure clarity to the reader regarding directionality

d. I don't think the word treated is the best fit here. Perhaps something related to intervention

e. I found the strengths are rather vague. I would appreciate for specificities

f. It seems this last paragraph informs beyond the objective. I wonder if authors could consider limiting it to the objective instead of advancing methods aspects

g. The use of correlate might not be correct here as association has been mentioned before

---

VERSION 1 - AUTHOR RESPONSE

Summary of Revisions

Major Comments

1. Page 6, line 36. For sake of clarity, I commend authors to find a better way to refer to anthropometric status. Currently at some instance is redundant or unclear the meaning. There is no reference to indicators such as stunting/wasting or underweight, that would make it much more clear to the readers.

We have clarified the references throughout the text, being more specific about the relevant indicators.

2. There are important gaps/uncertainties on how anthropometric measures were collected. It is very important that authors adhere to what WHO/UNICEF recommends. Please consult their 2019 guideline. For example, authors should provide details about valid anthropometric. I am very concerned with the statement about using 4cm to flag differences between two measures. Also, can authors inform what is the range for defining outliers?

Thank you for pointing this out. We will follow the 2019 WHO/UNICEF guidelines, and we have added details throughout the text, particularly throughout Section A.1.4. We have substantially narrowed the difference between multiple measures to trigger re-measurement (to >100g and >1cm). We have added the outlier range we will use during analysis. These latter two sections are also pasted below.

Added, Section A.1.4 (p18)

Biologically implausible values will be flagged and excluded prior to analysis following WHO-recommended cutoffs: height-for-age z-score < −6 or > +6; weight-for-age z-score < −6 or > +5; and weight-for-height z-score < −5 or > +5 [22]. Exclusion rates will be reported

Our primary results will use the initial height/length or weight measurement, following WHO guidelines. For robustness, we will also report estimates that average the initial estimate with a second estimate. In the case of substantial discrepancies (greater than 0.1 kg in weight or greater than 1cm in height/length), we will collect a third estimate and use the average of the two closest measurements.

3. Additionally, please inform more clearly about date of birth, a key component to calculate anthropometric variables. I strongly recommend authors include a data quality section to clarify the questions I have made.

In our data, we record the child's month and year (MM-YYYY) of birth, or, if available, day, month, and year (DD-MM-YYYY) of birth.

For our "baseline household sample" of children, we have the child's month and year of birth, as that was recorded at baseline in our cluster-randomized control trial (C-RCT). This information is verified during the midline survey, when parents are invited to bring their children for the assessment at the local daycare or community center.

For our "daycare sample" of children, parents have been asked to provide the date of birth of their child, in the DD-MM-YYYY format in the forms they return to the daycare along with their signed consent. The 2019 WHO/UNICEF guidelines recommend recording reported date of birth in situations where obtaining documents to verify the date might not be possible. We believe this is the case logistically, as child assessments done at daycares will be done with the consent of parents but not necessarily their presence.

As our data recording is done through surveyCTO, we have programmed it to note when children are 36 months or older, following the date of birth entry, so that the IDELA assessment can also be applied.The IDELA recommends recording child age, and, as noted in our protocol, we expect more than half of our sample (56–62%) to be 36 months and older and be administered the IDELA.

We have also expanded our "data monitoring" section into "data monitoring and quality," as recommended. It includes more detail on our data quality measures, such as team leads and/or management conducting spot checks and accompanying enumerators through sit-ins and using the in-built data quality monitoring checks from surveyCTO, which includes audio and text audits.

Added, Section 2.5 (p7)

Measurements of height and weight will follow the 2019 WHO/UNICEF guidelines and IDELA will be applied following the tool guidelines. Details are provided in Appendix A.1.4 and A.1.5, respectively.

Added, Section 2.5.1 (p7)

Our study sample is limited to children born in 2021 or later, and we will use child date of birth to calculate height-for-age z-scores as well as to apply the IDELA, which relies on child age in months. For our first group of children (those in the C-RCT baseline), we have the child's month and year of birth, as that was recorded in the C-RCT baseline. This information will be verified during our midline survey, when parents are invited to bring their children for the assessment.

For the group of children recruited through their daycare participation, parents will be asked to provide the date of birth of their children in the forms they return to the daycare along with their consent.

Added, Section 2.6.1 (p7–8)

For anthropometric measurements, data collection will follow the 2019 WHO/UNICEF guidelines [22]. IPA also uses the data quality monitoring checks built into surveyCTO.

4. Please state what type of equipments are being used along with their brands and accuracy.

We used a portable stadiometer approved by UNICEF which allows measuring length of children who cannot stand and height for children above 2 years who can stand. It has gradations at 0.1cm and accuracy of 0.2 cm. We use portable digital weighing scales (specifically, the Dawood 2005D) with 100g resolution.

Manufacturer-published accuracy tolerances were unavailable. However, we conducted field calibration checks before and during data collection.

We have noted this in the text:

Added, Section A.1.4, (p17–18):
For measuring child height/length, we use wooden height/length measuring boards approved by UNICEF, with a gradation of 0.1 cm and accuracy of 0.2 cm. Child height or length will be recorded in centimeters to the nearest decimal.

For child weight, we use a portable battery-powered digital scale with a range of 0 to 150 kilograms and a graduation of 100g, and we follow the 2019 WHO/UNICEF recommendations for calibration. Child weight will be recorded in kilograms.

5. A few other aspects deserve attention.
    a. Can authors write in full what C-RCT stands for in the abstract
    b. It would be preferable to state what are the validated instruments here. Perhaps remove less important details from the abstract. I see intro is rather lengthy
    c. I recommend the exposure is always listed before to ensure clarity to the reader regarding directionality

    We have implemented these suggestions, adjusted the title of the paper, and clarified the abstract

    d. I don't think the word treated is the best fit here. Perhaps something related to intervention

    We believe this comment refers to the abstract. We now word this sentence as "(2) examine differences in outcomes between children attending daycares in communities exposed to quality and control communities."

    e. I found the strengths are rather vague. I would appreciate for specificities

    We have made these strengths more specific.

    Revised text (p2)

    - Strength: We use well-established methods and tools to measure nutritional status and early child development : we conduct direct anthropometric assessment using standardized field protocols, and we

measure early child development outcomes using a validated, age-appropriate instrument (IDELA) administered by trained enumerators.

- Strength: Data collection targets children age 5 and under living in informal settlements across Kenya, a hard-to-reach population at high nutritional risk.
- Strength: Randomized assignment to differing levels of exposure to high-quality daycare provides a rigorous design to compare child outcomes while minimizing confounding from provider and household characteristics.

f. It seems this last paragraph informs beyond the objective. I wonder if authors could consider limiting it to the objective instead of advancing methods aspects

We believe you are referring to the last paragraph in Section 3.2. We revised this to restrict focus to the primary objectives. If we misunderstood, we're happy to address elsewhere.

Revised, Section 3.2 (p9)
Cross-sectional data gathered concurrently through caregiver and daycare firm surveys will produce a large data set to measure the relationship between early child development and nutritional status, as well as whether paid caregiving arrangements are associated with children's outcomes.

g. The use of correlate might not be correct here as association has been mentioned before

We have adjusted this in the text.
